# Are Genetic Risk Factors for Psychosis Also Associated with Dimension-Specific Psychotic Experiences in Adolescence?

**DOI:** 10.1371/journal.pone.0094398

**Published:** 2014-04-09

**Authors:** Dominika Sieradzka, Robert A. Power, Daniel Freeman, Alastair G. Cardno, Philip McGuire, Robert Plomin, Emma L. Meaburn, Frank Dudbridge, Angelica Ronald

**Affiliations:** 1 Centre for Brain and Cognitive Development, Birkbeck, University of London, London, United Kingdom; 2 King's College London, Medical Research Council Social, Genetic and Developmental Psychiatry Centre, Institute of Psychiatry, De Crespigny Park, London, United Kingdom; 3 Department of Psychiatry, University of Oxford, Oxford, United Kingdom; 4 Academic Unit of Psychiatry and Behavioural Sciences, University of Leeds, Leeds, United Kingdom; 5 Institute of Psychiatry, King's College London, London, United Kingdom; 6 Faculty of Epidemiology and Population Health, London School of Hygiene and Tropical Medicine, London, United Kingdom; University of Iowa Hospitals & Clinics, United States of America

## Abstract

Psychosis has been hypothesised to be a continuously distributed quantitative phenotype and disorders such as schizophrenia and bipolar disorder represent its extreme manifestations. Evidence suggests that common genetic variants play an important role in liability to both schizophrenia and bipolar disorder. Here we tested the hypothesis that these common variants would also influence psychotic experiences measured dimensionally in adolescents in the general population. Our aim was to test whether schizophrenia and bipolar disorder polygenic risk scores (PRS), as well as specific single nucleotide polymorphisms (SNPs) previously identified as risk variants for schizophrenia, were associated with adolescent dimension-specific psychotic experiences. Self-reported Paranoia, Hallucinations, Cognitive Disorganisation, Grandiosity, Anhedonia, and Parent-rated Negative Symptoms, as measured by the Specific Psychotic Experiences Questionnaire (SPEQ), were assessed in a community sample of 2,152 16-year-olds. Polygenic risk scores were calculated using estimates of the log of odds ratios from the Psychiatric Genomics Consortium GWAS stage-1 mega-analysis of schizophrenia and bipolar disorder. The polygenic risk analyses yielded no significant associations between schizophrenia and bipolar disorder PRS and the SPEQ measures. The analyses on the 28 individual SNPs previously associated with schizophrenia found that two SNPs in TCF4 returned a significant association with the SPEQ Paranoia dimension, rs17512836 (*p*-value = 2.57×10^−4^) and rs9960767 (*p*-value = 6.23×10^−4^). Replication in an independent sample of 16-year-olds (N = 3,427) assessed using the Psychotic-Like Symptoms Questionnaire (PLIKS-Q), a composite measure of multiple positive psychotic experiences, failed to yield significant results. Future research with PRS derived from larger samples, as well as larger adolescent validation samples, would improve the predictive power to test these hypotheses further. The challenges of relating adult clinical diagnostic constructs such as schizophrenia to adolescent psychotic experiences at a genetic level are discussed.

## Introduction

The notion of the psychosis continuum postulates that psychosis is a continuously distributed quantitative phenotype and disorders such as schizophrenia and bipolar disorder are its extreme manifestations [1] (see also [2]). Clinical psychotic symptoms include (but are not limited to) hallucinations, delusions, cognitive disorganisation, avolition and anhedonia [3] and can be measured in the general population [1]. ‘Psychotic experiences’ refers to these symptoms when assessed as experiences across the full range of severity in the general population.

Some evidence suggests that early psychotic experiences are a risk marker for later development of a psychotic disorder [4],[5], although most individuals with psychotic experiences in adolescence do not go on to develop psychotic disorders. High scores on psychotic experiences in childhood (age 11) were shown to be an indication of an increased risk of developing a psychotic disorder later in life (age 26)[4]. However, it is typically not until adolescence/early adulthood that psychotic symptoms first emerge [6] and the association between psychotic experiences and a number of psychiatric disorders strengthens [7]. In a study conducted by Kelleher et al. [7] prevalence of psychotic experiences have been shown to decrease over time, from 21% in early adolescence (11–13 year olds) to 7% in mid-adolescence (13–16 year olds). In contrast to this, the predictive power of these psychotic experiences for a number of psychiatric disorders strengthened with an increase in age. These findings highlight the potential value of studying psychotic experiences in mid-adolescence.

A number of studies have shown that, consistent with adult schizophrenia, psychotic experiences in adolescence show a multidimensional factorial structure [8]–[14]. Although the number and content of the reported dimensions varies across studies due to the measures and statistical analyses used, most report at the very least three dimensions of Positive, Negative and Disorganisation. Ronald et al. [14] created a quantitative dimension-specific assessment of positive, cognitive and negative psychotic experiences in adolescence, used in the present study, which was found to show a six-dimensional structure (Paranoia, Hallucinations, Cognitive Disorganisation, Grandiosity, Anhedonia and Negative Symptoms).

In terms of the role of genetic influences on adolescent psychotic experiences, published twin studies report heritabilities of psychotic experiences in adolescence to range from 33–57% depending on dimension [13],[15],[16]. Heritabilities of the six dimensions used here ranged from 15–59% (unpublished). The highest heritabilities were noted for the Negative Symptoms (59%) and Paranoia (50%) dimensions of psychotic experiences, with the lowest heritabilities being for Hallucinations (15% for males and 32% for females).

Findings from twin studies suggest that heritabilities of psychotic disorders are higher than for adolescent psychotic experiences. Estimates of heritability of both schizophrenia and bipolar disorder have been shown to be ∼80% [17]–[19] and the bivariate heritability between the two disorders to be 63% [20]. Common genetic variants have been shown to play an important role in the aetiology of both disorders [21]. GWAS that investigate common single nucleotide polymorphisms (SNPs) have identified several SNPs in genic and non-genic regions that associate with schizophrenia risk (reviewed in [22]). A review of these loci [22] refers to 16 reliably replicated genes/regions that harbour SNPs that are GWAS significant. A different approach is to use a polygenic risk score (PRS), which aggregates genome-wide individual SNPs into a single score. Currently available PRS from the Psychiatric Genomics Consortium account for 23% of variation in schizophrenia liability [23] and a much smaller but significant proportion of ∼3% in bipolar disorder liability [24]. PRS can be used as predictors of phenotypes in other samples.

In terms of the genetic relationship between adolescent psychotic experiences and adult psychotic disorders, currently the genetic correlation (the degree of overlapping genetic influences) between adolescent psychotic experiences and clinical psychosis remains unknown. Here we hypothesise that if genes influencing risk for liability to psychosis are common in the general population, and if schizophrenia and bipolar disorder lie on a phenotypic continuum with psychotic experiences assessed dimensionally, schizophrenia and bipolar disorder identified genetic risk variants will also be associated with psychotic experiences.

Derks et al. [25] were the first to explore the correlation between the schizophrenia PRS and a dimensional quantitative measure of schizophrenia symptoms using a case-control design (N = 314 cases, 148 controls). The authors identified a five-dimensional structure of psychosis but found no significant correlation between any dimension and the schizophrenia PRS, after accounting for case-control status. In contrast to this, Fanous et al. [26], identified a three-dimensional structure and with a sample size over twenty-times the magnitude of the previous study, found schizophrenia negative/disorganised symptom dimension PRS to be a significant predictor of the PGC schizophrenia case-control status (*r* ^2^ = 0.0005, *p*-value = 0.007). In a separate analysis the authors also found the negative/disorganised symptom dimension to be correlated with the PGC schizophrenia PRS (*p*-value = 0.03).

To date, one study has explored the association between psychotic experiences in the general population and schizophrenia associated genetic risk variants [27]. Psychotic experiences were assessed as a single categorical construct, where presence of psychotic experiences was defined as presence of any one of a number of different positive psychotic experiences. With a sample of 3,483 individuals assessed when 12 and 18 years old, the authors reported that on average individuals with psychotic experiences had higher schizophrenia PRS than those without psychotic experiences, however the lowest *p*-value noted did not reach significance (*p*-value = 0.134 at p_T_<0.3). The respective odds ratio per standard deviation increase in score was 1.08 for the schizophrenia PRS (calculated based on the results from the PGC GWAS stage-1 mega-analysis), which the authors regarded as ‘very weak evidence’ for an association with psychotic experiences.

The present study adds to this recent work by using *both* the schizophrenia and bipolar disorder PRS and testing for their predictiveness with quantitatively-measured specific psychotic experiences. The present study includes separate scales for different types of positive psychotic experiences (paranoia, hallucinations, grandiosity and delusion), as well as including cognitive disorganisation and negative psychotic experiences, which has not been done before. The measures were quantitative and therefore captured varying severity of manifestation of psychotic experiences across the population. For example, paranoia was assessed in terms of how frequently individuals had paranoid thoughts and items ranged in severity from mild suspicions that others have an interest in the person all the way to fears of conspiracies.

In *Approach 1*, estimates of variance explained in dimension-specific psychotic experiences in adolescence using schizophrenia and bipolar disorder PRS(s) were derived. For both the schizophrenia PRS and the bipolar disorder PRS, we hypothesised that the PRS would be separately associated with each of the six dimensions of psychotic experiences. We further hypothesised that the associations would be positive. It was expected that the PRS scores would explain a smaller but significant proportion of variance in adolescent psychotic experiences than they did for the liability to schizophrenia and bipolar disorder. In *Approach 2*, 33 selected SNPs from 16 genes/regions previously identified as influencing risk for diagnosed schizophrenia [22] were tested for association with quantitative dimension-specific psychotic experiences in adolescents. For each dimension of psychotic experiences in adolescence, we hypothesised that the selected risk variants would also be associated with that dimension; we regarded these as separate hypotheses to be tested individually, without adjustment for multiplicity. It was expected that while some associations would be specific to certain dimensions and others would show pleiotropic effects (i.e. be associated with multiple different types of psychotic experiences), all associations would be positive. In *Approach 3*, a composite schizophrenia SNP score made up of the selected SNPs from Approach 2 was created to estimate the variance explained in quantitative dimension-specific psychotic experiences in adolescence. It was hypothesised that the composite schizophrenia SNP score would be a significant predictor of quantitative dimension-specific psychotic experiences in adolescence and that it would explain a small proportion of variance. Finally, significant findings from Approaches 2 and 3 were tested for replication in an independent population-based sample of adolescents (as used in [27]).

## Methods

### Participants

#### TEDS, Validation Sample

Individuals who participated in the current study were drawn from the Twins Early Development Study (TEDS). TEDS is a longitudinal general population sample of twins born in England and Wales between 1994 and 1996 [28]. TEDS originally recruited 13,488 families, who responded with a written consent form.

The current study forms part of the Longitudinal Experiences And Perceptions (LEAP) project, which investigates the aetiology of psychotic experiences in adolescence. For the purposes of this study families were not contacted if they had withdrawn from TEDS, had never returned any data or had known address problems. This resulted in 10,874 TEDS families being contacted and invited to participate in LEAP. Of those, 5,076 (46.7%) parents and 5,059 (46.5%) twins provided data on quantitative dimension-specific psychotic experiences at age 16 years (Mean = 16.32 years; SD = 0.68). Participants were excluded based on lack of consent at first contact or for the present study, presence of severe medical disorder(s) including autism spectrum disorder, lack of zygosity information or experience of severe perinatal complications.

DNA extracted from buccal cheek swabs from 4,440 children from TEDS were sent to Affymetrix Santa Clara, California, USA to be individually genotyped on the AffymetrixGeneChip 6.0 SNP genotyping platform as part of the TEDS Wellcome Trust Case Control Consortium 2 (WTCCC2) study of reading and mathematical abilities (see http://www.wtccc.org.uk/ccc2/wtccc2_studies.shtml). In total, 3,665 samples were successfully hybridized to AffymetrixGeneChip 6.0 SNP genotyping arrays. Of the genotyped individuals, 513 were excluded based on one or more of the following parameters: low call rate or heterozygosity outliers (377), atypical population ancestry (59), sample duplication or relatedness to other sample members (83), unusual hybridization intensity (9), gender mismatches (13), and having less than 90% of genotypes called identically on the genome-wide array and Sequenom panel (54) [29]. The final sample of 3,152 individuals comprised 1,446 males and 1,706 females.

Phenotypic data on psychotic experiences was available for 2,152 of the 3,152 genotyped individuals and limited to those who were unrelated and of white background. The final sample was 43% male.

#### ALSPAC, Replication Sample

The Avon Longitudinal Study of Parents and Children (ALSPAC) [30] core cohort comprised of 14,541 pregnancies with an expected delivery date between 1^st^ April 1991 and 31^st^ December 1992 (www.alspac.bris.ac.uk). When the oldest children of the 14,541 pregnancies were 7 years old additional pregnancies that failed to enrol at first attempt were added. This resulted in a total sample size of 15,247 pregnancies (15,458 fetuses). Of the 15,458 fetuses, 14,775 were live births and 14,701 were alive at 1 year of age.

9,912 children from ALSPAC were individually genotyped on the Illumina HumanHap550 quad genome-wide SNP genotyping platform. Of the genotyped individuals, some were excluded based on one or more of the following parameters: incorrect gender assignments (61), minimal or excessive heterozygosity (375), disproportionate levels of individual missingness (15), evidence of cryptic relatedness (1182), and non-European ancestry (734) [31]. The resulting sample comprised 8,365 genotyped individuals.

In total 4,458 children in ALSPAC provided data on psychotic experiences at age 16 years (M = 16.68 years; SD = 0.24). Genotypic data was available for 3,427 of those individuals and limited to unrelated individuals of European ancestry. The final sample was 42% male. Note that the study website contains details of all the data that is available through a fully searchable data dictionary (http://www.bris.ac.uk/alspac/researchers/data-access/data-dictionary/).

Ethical considerations prevented us from publicly depositing the raw genotypic and phenotypic data but it can be made available in a suitable form on request from TEDS and ALSPAC.

### Ethics Statement

Twins Early Development Study (TEDS) and consent procedure were approved by the Institute of Psychiatry ethics committee (ref: 05/Q0706/228). The Avon Longitudinal Study of Parents and Children (ALSPAC) and consent procedure ethical approval was obtained from the ALSPAC Ethics and Law Committee (IRB 00003312) and the Local Research Ethics Committees. Informed written consent was obtained for both studies.

### Measures

#### Specific Psychotic Experiences Questionnaire (SPEQ)

In TEDS, dimension-specific psychotic experiences were assessed using the Specific Psychotic Experiences Questionnaire (SPEQ;[14]). SPEQ includes five self-report subscales (Paranoia, Hallucinations, Cognitive Disorganisation, Grandiosity and Anhedonia) and one parent-rated subscale (Parent-rated Negative Symptoms). SPEQ was based on existing measures that were adapted specifically for use with adolescents. Items were placed into separate subscales based on the results of principal component analysis [14].

*Paranoia* subscale comprised 15 items rated on a 6-point scale (not at all; rarely; once a month; once a week; several times a week; daily) and the total scale ranged from 0 to 75. *Hallucinations* subscale of SPEQ comprised nine items measured on a 6-point scale (not at all; rarely; once a month; several times a week; once a week; daily) and the total scale ranged from 0 to 45. *Cognitive Disorganisation* subscale of SPEQ comprised eleven items measured on the scale of yes/no responses with the range of total scores from 0 to 11. *Grandiosity* subscale consisted of eight items measured on a 4-point scale (not at all; somewhat; a great deal; completely) and the total scale ranged from 0 to 24. *Anhedonia* subscale consisted of 10-items asking about hedonia rated on a 6-point scale (very false for me; moderately false for me; slightly false for me; slightly true for me; moderately true for me; very true for me) and the total scale ranged from 0 to 50. Anhedonia scale was reversed so that higher scores signified more Anhedonia. Finally, *Parent-reported Negative Symptoms* subscale was made up of 10- items rated on a 4-point scale (not at all true; somewhat true; mainly true; definitely true) and the total scale ranged from 0 to 30.

SPEQ subscales show good to excellent internal consistency (Cronbach's α ranged from .77 to .93) and test re-test reliability (*r* = .65 to .74 across an average 9-month interval; all *p*<0.001). The subscales also show good content and construct validity [14]. The derivation of items, and full information on reliability and validity is also available elsewhere [14].

#### Psychosis-Like Symptoms measure (PLIKS-Q)

In the replication sample psychotic experiences were assessed using the Psychosis-Like Symptoms Questionnaire (PLIKS-Q; [32]). The ALSPAC sample was selected for the purposes of replication of the findings from the validation sample based on the similar age and agreement between the PLIKS-Q and SPEQ subscales [14]. The phenotypic correlations between PLIKS-Q quantitative score and positive SPEQ subscales were significant, positive, and moderate to high in magnitude: Hallucinations *r* = .60, Paranoia *r* = .48, Cognitive Disorganisation *r* = .41, Grandiosity *r* = .27 (all *p*<0.001).

PLIKS-Q quantitative score was computed based on responses to 10 items rated on a 3-point scale (yes, definitely; yes, maybe; no, never) that assesses positive psychotic experiences (hallucinations, delusions and thought interferences). Originally “no, never” responses were coded with the highest numerical value hence PLIKS-Q total quantitative score was reversed so that higher scores signified more psychotic-like symptoms. At least 5 responses were required in order to calculate the total score and the scale ranged from 1-30.

## Statistical Analyses

### Phenotypic Analyses

Descriptive statistical analyses were performed in SPSS for Windows (version 18.0).

#### Scale Transformation

Due to the moderate skew of the psychotic experiences scales as measured by SPEQ the data were transformed using log_10_(1+variable) formulae for the polygenic risk analyses. This transformation was selected because it was considered most suitable for previously conducted analyses on these measures and to enable direct comparison between analyses reported elsewhere (unpublished) and variance explained by the polygenic risk scores (PRS). For comparison polygenic risk analyses were also performed using the van der Waerden transformation and the pattern of results remained unchanged.

For the single SNP analyses the psychotic experiences scales as measured by SPEQ and PLIKS-Q were transformed using van der Waerden's transformation [33] to normalise the data, which works by converting ranked data to the quantiles of the standard normal distribution.

#### T-tests

Two-tailed independent *t*-tests were conducted to describe mean differences between males and females. Where Levene's test was significant, *p*-values for corrected degrees of freedom (*df*) were reported.

### Approach 1

#### Polygenic Risk Scores (PRS)

In the current study, Psychiatric Genomics Consortium (PGC) schizophrenia [34] and PGC bipolar disorder [24] stage-1 GWAS mega-analysis full results were used to create two PRS; one for schizophrenia and one for bipolar disorder. First, linkage disequilibrium (LD) clumping at *p*-value thresholds of 0.01, 0.05, 0.1, 0.2, 0.3, 0.4, 0.5, and 1.00 was performed on 943,564 SNPs with high imputation information (≥0.90) in PLINK version 1.07 [35]; http://pngu.mgh.harvard.edu/purcell/plink/), an open-source whole genome association analysis toolset. MHC region (26–33 Mb) was excluded based on a complex LD structure in this region [34]. The procedure undertaken pruned to *r* ^2^ = 0.25 within 200 kb windows. The SNPs in the TEDS sample were also required to have a minor allele frequency (MAF) >0.02; genotyping >0.90; and Hardy-Weinberg Equilibrium *p*>1×10^-6^. PRS were then calculated in PLINK for each genotyped individual in the TEDS sample by summing the risk alleles weighted by the estimated log of odds ratios obtained from the PGC results. PRS were calculated for eight *p*-value thresholds (p_T_). Numbers of SNPs per threshold are summarised in Tables S1 and S2 in File S1.

#### Polygenic risk analyses

Linear regression analyses were performed in SPSS for Windows (version 18.0) with PRS scores as predictors of the six SPEQ subscales. The significance threshold for the polygenic risk analyses was set to *p*<0.05 (one-tailed) as correlations between different *p*-value thresholds, p_T_ 0.01 to 1.00, range from .62 to .99. To control for population stratification, principal component analyses (PCA) were performed and using the Tracy-Widom test eight principal components (PCs; *p*<0.05) were identified as covariates and included in the model. Full details of the PCA analyses can be found in [29].

### Approach 2

#### Selected SNPs for the single SNP analyses

SNPs previously identified as genetic risk variants for schizophrenia significant at genome-wide threshold of at least *p*≤5×10^-8^ were chosen. The initial selection process was informed by the review of the genome-wide association studies (GWAS) of schizophrenia findings undertaken by Bergen and Petryshen [22]. Further examination of the original publications was required in order to identify the actual SNPs and corresponding risk alleles from the genes and *p*-values cited in the review [22]. This resulted in identification of 10 additional SNPs that met our inclusion criteria and the final selection of 33 genetic risk variants significantly associated at *p*≤5×10^−8^ with schizophrenia. A summary of selected SNPs is provided in Table S3 in File S1. Three of these variants were significant based on joint analysis of schizophrenia and bipolar disorder. These were *rs4765905* (CACNA1C; chromosome 12; *p* = 7.01×10^−9^), *rs10994359* (ANK3; chromosome 10; *p* = 2.45×10^−8^), and *rs2239547* (ITIH3-ITIH4 region; chromosome 3, *p* = 7.83×10^−9^) [34].

### Proxies & Quality Control (QC) in the single SNP analyses

#### TEDS, Validation Sample

Six of the 33 selected SNPs were neither genotyped nor imputed in the validation dataset and a proxy SNP in linkage disequilibrium (LD) with that candidate SNP (*r* ^2^>.8; D' = 1) was identified based on observed patterns in SNAP [36]. Stringent quality control (QC) as per the genotyped data was performed. QC filters for the genotypic data required that genotyping was above 95% complete for each individual and that each SNP had above 90% genotyping. SNPs that were not in Hardy-Weinberg Equilibrium were excluded (values below *p*<1×10^−6^), as were SNPs with a MAF of less than 2%. As a result five of the 33 selected SNPs (rs6913660, rs13194053, rs3800316, rs6932590, and rs6904071) were excluded from further analyses. The average call rate per individual post exclusion of the five SNPs was 99.81%. The results of the QC analyses are summarised in Table S4 in File S1. QC analyses were performed using PLINK.

#### Single SNP analyses

Allelic and genotypic association analyses were undertaken between all SPEQ subscales and the selected SNPs using the linear regression function in PLINK. Allelic association analyses were performed using an additive linear regression model. Genotypic association analyses were performed using a two degree of freedom joint test of additivity and dominance deviation. Age and sex were included as covariates in this study.

The corrected *p*-value significance threshold using Bonferroni adjustment was set to *p*<0.0008 (0.05/(33×2); one-tailed), where 0.05 represents nominal significance cut-off, 33 represents the number of selected SNPs before QC, and 2 represents types of genetic tests conducted (i.e. allelic and genotypic). The Bonferroni correction is conservative as it assumes that all tests performed are independent of one another and could therefore result in overcorrection and potential false negatives. For this reason, for significant findings an adaptive permutation approach implemented in PLINK was also employed as it allows the correlational structure between SNPs to be maintained while manipulating the genotype-phenotype relationship to generate appropriate empirical significance levels (p_EMP_). Statistical package R (http://www.r-project.org/) and LocusZoom [37] were used for graphical representation of all significant results.

### Approach 3

#### Schizophrenia composite SNP Score

The composite SNP score was created by summing unweighted risk alleles (as per the original publications) of the 28 SNPs of the selected 33 that passed QC. Linear regressions were performed to estimate variance explained by the composite SNP score in quantitative psychotic experiences in the validation sample.

### Replication

#### ALSPAC, Replication Sample

Significant SNPs from *Approach 2* were replicated in the ALSPAC sample. rs17512836 was not available in the ALSPAC dataset, hence rs17597926 and rs17527346 were identified as proxies (*r* ^2^ = . 83; D' = 1) based on observed patterns in SNAP [36]. Two proxies rather than one were selected to ensure that replication was still possible in case of one of the SNPs failing quality control (QC). Stringent QC was performed on the SNPs under investigation and the QC filters were set to match those from the validation sample. Neither SNPs nor individuals required exclusion based on the set QC filters. The average call rate per individual was 99.98%. The results of the QC analyses are summarised in Table S5 in File S1.

Allelic and genotypic association analyses were undertaken between the PLIKS-Q measure and the selected SNPs using the linear regression function in PLINK. Age and sex were included as covariates.

## Results

### Descriptive Statistics

Descriptive statistics for the SPEQ and PLIKS-Q measures are summarised in Table 1. SPEQ showed significant phenotypic mean sex differences across four subscales of psychotic experiences (*p*<0.05). Females scored significantly higher than males on Cognitive Disorganisation and males scored significantly higher than females on Grandiosity, Anhedonia and Parent-rated Negative Symptoms (*p*<0.001). Paranoia and Hallucinations subscales showed no significant mean differences between sexes although there was a trend for females to report more experiences. A summary of results is presented in Table 2. PLIKS-Q (*t*(1,3310.21) = 8.53, *p*<0.001) showed significant mean sex differences with females scoring higher than males.

**Table 1:** Descriptive statistics for the six dimensions of psychotic experiences as measured by SPEQ and PLIKS-Q.

	Paranoia	Hallucinations	Cognitive Disorganisation	Grandiosity	Anhedonia	Parent-rated Negative Symptoms	PLIKS-Q
N	2133	2138	2133	2136	2134	2140	3427
Mean	11.99	4.54	3.81	5.15	16.03	2.69	1.95
Median	10.00	2.00	3.00	4.00	15.00	1.00	1.00
SD	10.18	5.79	2.83	4.22	7.66	3.66	1.83
Variance	103.70	33.53	8.00	17.82	58.71	13.36	3.34
Observed Range	0–71	0–42	0–11	0–24	0–46	0–28	1–16
Skewness	1.46	1.95	0.52	1.12	0.57	2.38	2.89

Note. SD, standard deviation.

**Table 2:** Mean sex differences on psychotic experiences.

	Male		Female				
	M	SD	M	SD	*t*	*df*	*p*
Paranoia	11.85	9.90	12.10	10.40	0.45	1, 2131	.656
Hallucinations	4.51	5.86	4.56	5.74	0.78	1, 2136	.438
Cognitive Disorganisation	3.22	2.63	4.26	2.89	8.65	1,206.54	<.01
Grandiosity	5.80	4.44	4.66	3.97	6.62	1, 2134	<.001
Anhedonia	18.10	7.61	14.47	7.32	11.13	1, 2132	<.001
Parent-rated Negative Symptoms	3.06	3.88	2.42	3.46	4.91	1, 2138	<.001
PLIKS-Q	1.71	1.61	2.13	1.95	8.53	1,3310.21	<.001

Note. M = Mean; SD = standard deviations; df = degrees of freedom. Raw scores provided. Paranoia, Hallucinations, Grandiosity and Parent-rated Negative Symptoms were transformed prior to statistical testing.

### Approach 1 results

Descriptive statistics for the schizophrenia PRS and bipolar disorder PRS are presented in Tables S6 and S7 in File S1. None of the six dimensions of psychotic experiences (Paranoia, Hallucinations, Cognitive Disorganisation, Grandiosity, Anhedonia, and Parent-rate Negative Symptoms) showed significant positive associations with either schizophrenia or bipolar disorder PRS across all thresholds at *p*<0.05 (one-tailed). The summary of results for the schizophrenia and bipolar disorder PRS at p_T_ = 0.5 are presented in Tables 3 and 4 respectively. Full results for all thresholds per psychotic experiences dimension are presented in Tables S8 to S19 in File S1.

**Table 3:** Summary of results for schizophrenia PRS as a predictor of SPEQ subscales at p_T_ = 0.5.

SPEQ Subscale	β	95% C.I.	t	*p*-value	R^2^	N	df
Paranoia	−375.64	−1008.33–257.04	−1.16	0.122	.001	2157	9, 2147
Hallucinations	63.22	−610.29–736.72	0.18	0.427	.000	2162	9, 2152
Cognitive Disorganisation	−195.63	−4640.01–4248.75	−0.09	0.466	.000	2157	9, 2147
Grandiosity	−104.72	−627.64–418.21	−0.39	0.329	.000	2160	9, 2150
Anhedonia	−16719.75	−28744.46–(–4695.03)	−2.27	0.003	.003	2158	9, 2148
Parent-rated Negative Symptoms	−554.82	−1128.93–19.28	−1.89	0.029	.002	2162	9, 2152

Note. β, unstandardized beta value; C.I., confidence intervals; t, t-statistic; N, sample size; df, degrees of freedom; SPEQ Paranoia subscale was log transformed; 8 PCs were included as covariates.

**Table 4:** Summary of results for bipolar disorder PRS as a predictor of SPEQ subscales at p_T_ = 0.5.

SPEQ Subscale	β	95% C.I.	t	*p*-value	R^2^	N	df
Paranoia	−440.61	−1008.57–127.36	−1.52	0.064	.001	2157	9, 2147
Hallucinations	−273.88	−878.88–331.12	−0.89	0.188	.000	2162	9, 2152
Cognitive Disorganisation	−1256.09	−5243.68–2731.51	−0.62	0.269	.000	2157	9, 2147
Grandiosity	−243.03	−712.87–226.82	−1.01	0.156	.000	2160	9, 2150
Anhedonia	−9194.52	−19999.30–1610.27	−1.67	0.048	.001	2158	9, 2148
Parent-rated Negative Symptoms	−204.39	−720.71–311.92	−0.78	0.219	.000	2162	9, 2152

Note. β, unstandardized beta value; C.I., confidence intervals; t, t-statistic; N, sample size; df, degrees of freedom; 8 PCs were included as covariates.

### Approach 2 results

#### Single SNP analyses

**Figure 1:**
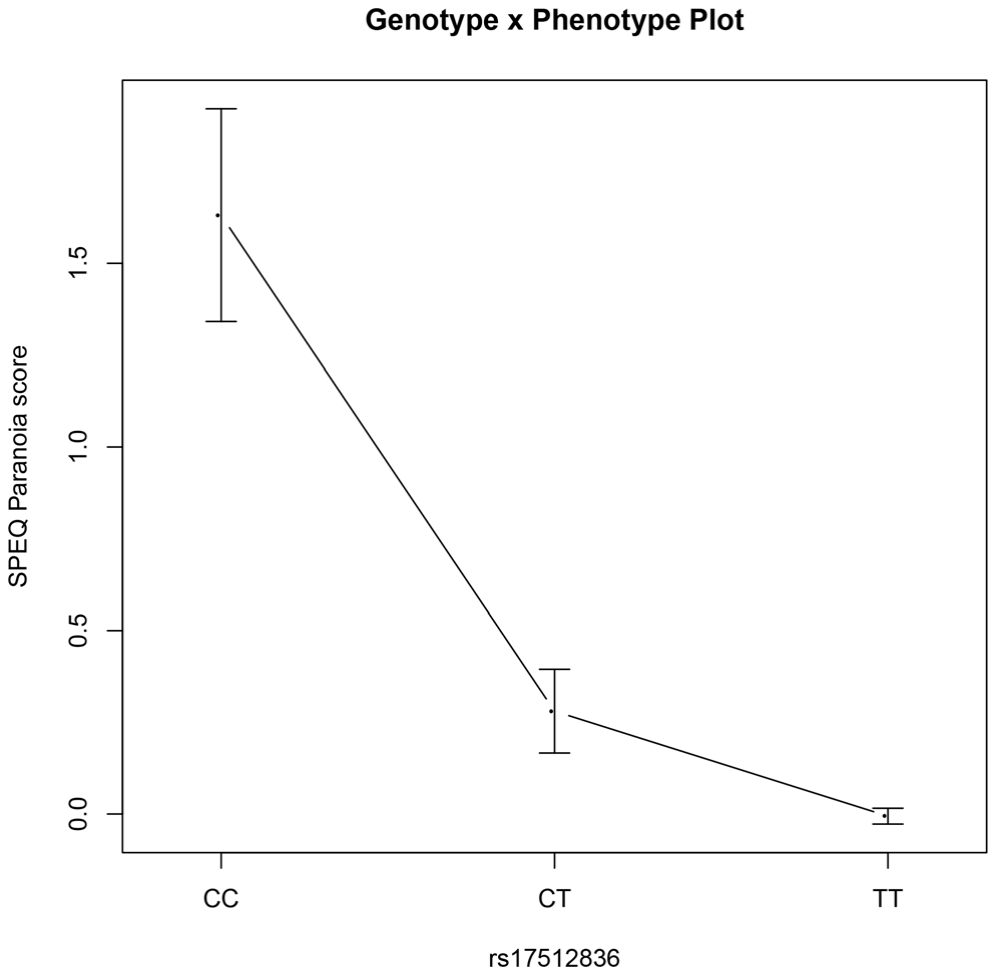
van der Waerden transformed mean SPEQ Paranoia scores plotted by rs17512836 genotypes.

**Figure 2:**
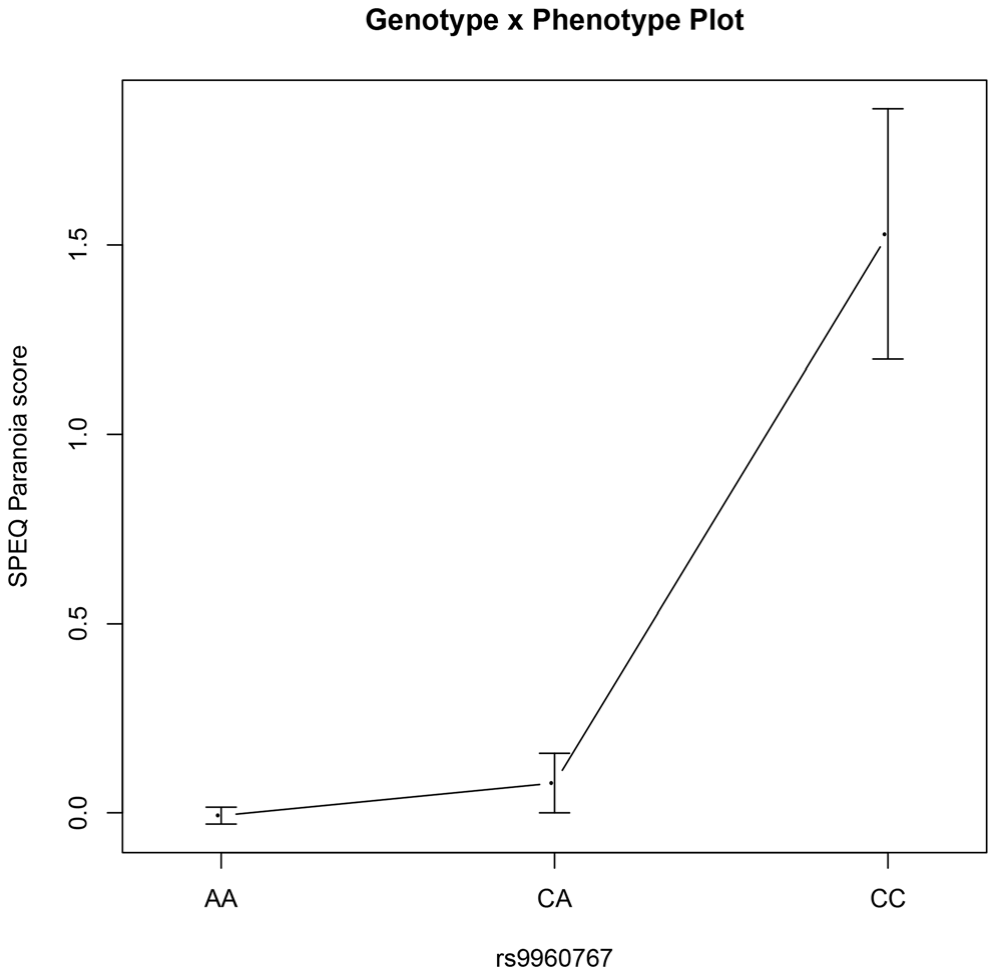
van der Waerden transformed mean SPEQ Paranoia scores plotted by rs9960767 genotypes.

The summary of top results (*p*<0.05; one-tailed) for allelic and genotypic association analyses are presented in Table 5. (Full results can be found under supplementary materials Table S20 in File S1). A total of twenty-eight out of thirty-three SNPs previously associated with schizophrenia were tested for associations with dimension-specific psychotic experiences. Fourteen out of twenty-eight selected SNPs showed nominally significant associations at *p*<0.05 with different dimension-specific quantitative assessments of psychotic experiences but each failed to meet statistical significance post correction for multiple testing (*p*<0.0008; one-tailed). The strongest association was observed between the SPEQ Paranoia psychotic experiences subscale and rs17512836 in TCF4 with C identified as the risk allele. Both allelic association under an additive model (β = 4.17; *p*_rawdata_ = 3.86×10^−5^; p_EMP_ = 1.16×10^−4^; β = 0.35; *p*_transformeddata_ = 2.57×10^−4^; p_EMP_ = .001) and genotypic association (*p*_rawdata_ = 2.09×10^−5^; p_EMP_ = 0.006; *p*_transformeddata_ = 2.60×10^−4^; p_EMP_ = 0.005) were significant for rs17512836 and Paranoia. In addition, association between rs9960767 in TCF4 and Paranoia was significant in the genotypic test (*p*_rawdata_ = 4.44×10^−5^; p_EMP_ = 9.33×10^−4^; *p*_transformeddata_ = 6.23×10^−4^; p_EMP_ = 0.005). Figures 1 and 2 illustrate the above results in terms of transformed mean scores for the SPEQ Paranoia psychotic experiences subscale and the three genotypes per SNP.

**Table 5:** Summary results of allelic & genotypic association analyses for transformed SPEQ data with *p*-values <0.05 (adjusted for sex and age).

						Allelic Association				Genotypic Association
	SNP	Chr	Position	Allele	N	Beta	t-Stat	SE	ADD *p*-value	Geno_2DF *p*-value
Paranoia	rs1625579	1	98275522	T	2111	0.08	2.02	0.04	0.022	0.058
	rs1344706	2	185486673	C	2115	0.06	2.09	0.03	0.019	0.055
	rs4765905	12	2219845	C	2133	0.05	1.58	0.03	0.058	0.045
	rs9960767	18	51306000	C	2084	0.16	2.12	0.08	0.017	6.23×10^−4^
	rs17512836	18	51345959	C	2126	0.35	3.48	0.1	2.57×10^−4^	2.60×10^−4^
Hallucinations	rs10489202	1	166169703	T	2137	−0.02	−0.74	0.03	0.231	0.047
	rs9960767	18	51306000	C	2089	−0.02	−0.24	0.07	0.405	0.010
	rs17512836	18	51345959	C	2131	0.16	1.71	0.1	0.044	0.011
Cognitive	rs3800307	6	27293771	A	2133	−0.02	−0.66	0.03	0.253	0.005
	rs4452638	6	27337244	A	2133	−0.07	−1.99	0.04	0.024	0.068
	rs6938200	6	27339129	G	2133	0.04	0.86	0.04	0.195	0.016
	rs2021722	6	30282110	C	1953	0	0	0.04	0.5	0.008
	rs3131296	6	32280971	T	1947	0.03	0.66	0.04	0.255	0.02
	rs12807809	11	124111495	T	2108	0.07	1.68	0.04	0.047	0.096
	rs17512836	18	51345959	C	2126	0.23	2.34	0.1	0.01	0.021
Grandiosity	rs1344706	2	185486673	C	2118	−0.01	−0.27	0.03	0.395	0.01
Anhedonia	rs10503253	8	4168252	A	2115	0.06	1	0.06	0.159	0.031
Neg. Sympt.	rs1625579	1	98275522	G	2118	−0.07	−1.84	0.04	0.065	0.033
	rs10503253	8	4168252	A	2121	−0.08	−2.34	0.03	0.01	0.028
	rs548181	11	124966919	A	2133	0.08	1.86	0.04	0.032	0.076

Note: Chr, chromosome; Position, positions based on Human Genome Build 36; Allele, risk allele; Beta, regression coefficient; ADD, additive linear regression model; GENO_2DF, two degrees of freedom joint test of additivity and dominance deviation (it does not assume a linear relationship); Neg. Sympt., Parent-rated Negative Symptoms

The regional association plot in Figure S1 in File S1 provides a more in-depth view of the flanking region of 400 kb around rs17512836. The plot illustrates the associated region in the context of local patterns of LD. Specifically, the figure highlights four SNPs aside from rs17512836 that have a *p*≤7.57×10^−4^. The strongest associated SNP (rs41437147, *p* = 8.47×10^−5^; two-tailed) is an imputed SNP, however, two of the four SNPs with a *p*≤7.57×10^−4^ in this region were genotyped. rs35969244 and rs41515848 with a *p* = 1.14×10^−4^ (two-tailed) and *p* = 1.71×10^−4^ (two-tailed) respectively. This confirms that the signal in this region is not based purely on imputed SNPs.

### Approach 3 results

The schizophrenia unweighted composite SNP score of 28 SNPs was not a significant predictor of any of the SPEQ psychotic experiences subscales (*p*-values ranged from 0.310–0.886). Summary results of these analyses are presented in Table 6. For comparison purposes a composite score based on summing of *weighted* risk alleles (based on published effect sizes) was also created but it did not yield any significant results (not shown).

**Table 6:** Results of regression analysis with the schizophrenia composite SNP score as a predictor of the SPEQ measures (adjusted for sex and age and performed on the transformed data).

	β	SE	*t*-Stat	*p*-value	C.I.	R^2^
Paranoia	0.13	0.54	0.35	.730	−0.592–0.845	.002
Hallucinations	−0.35	0.34	−1.02	.310	−1.025–0.33	.001
Cognitive Disorganisation	−0.34	0.35	−0.96	.337	−1.026–0.35	.036
Grandiosity	−0.14	0.35	−0.38	.701	−0.826–0.556	.021
Anhedonia	−0.12	0.36	−0.34	.737	−0.816–0.577	.057
Parent-rated Negative Symptoms	−0.05	0.33	−0.14	.886	−0.694–0.599	.015

Note: β, Beta; SE, standard error; C.I., confidence interval.

### Replication results

Replication of findings from Approach 2 was attempted using the ALSPAC sample in relation to rs9960767 and rs17512836 that reached significance in association with the SPEQ Paranoia subscale in TEDS.

In total three SNPs (two SNPs were used as a proxy for rs17512836) previously associated with schizophrenia and the Paranoia SPEQ measure were tested for association with a quantitative assessment of positive symptoms, PLIKS-Q in the ALSPAC sample. The analyses did not yield any significant results at *p*<0.05. These are summarised in Table 7.

**Table 7:** Results of Allelic & Genotypic Association Analyses for PLIKS-Q Transformed Data (adjusted for sex and age)

						Allelic Association			Genotypic Association
SNP	Chr	Position	Allele	N	Beta	*t*-Stat	SE	ADD *p_transforemed_*-value	Geno_2DF *p_transformed_*-value
rs9960767	18	51306000	C	3419	0.01	0.15	0.04	0.439	0.447
rs17597926*	18	51356936	A	3427	0.05	0.81	0.06	0.209	0.349
rs17527346*	18	51448989	C	3427	0.05	0.82	0.06	0.205	0.345

Note: Chr, chromosome; Allele, risk allele; Beta, regression coefficient; ADD, additive linear regression model; GENO_2DF, two degrees of freedom joint test of additivity and dominance deviation (it does not assume a linear relationship); * proxies for rs17512836.

## Discussion

To our knowledge, this is the first study to investigate an association between quantitative dimension-specific psychotic experiences in adolescence and common genetic variants previously identified as risk factors for schizophrenia and bipolar disorder. It joins one other genetic study of psychotic experiences in adolescence, which reported weak evidence for an association between categorically defined positive psychotic experiences in adolescents in the general population and a schizophrenia polygenic risk score, and no significant associations between individual schizophrenia-associated SNPs and adolescent psychotic experiences [27].

### Approach 1: schizophrenia and bipolar disorder polygenic risk analyses

The schizophrenia and bipolar disorder polygenic risk score analyses, using the full results of the PGC stage-1 GWAS mega-analysis [24],[34], yielded no significant positive associations with dimension-specific psychotic experiences.

There are several plausible explanations for our lack of findings. First, it is possible that the schizophrenia and bipolar disorder PRS are significant predictors of other dimensions of psychotic experiences (e.g. mania) that were not assessed in the current study. However the SPEQ measure provided a fairly comprehensive assessment of specific positive, cognitive and negative psychotic experiences. Second, the phenotypes from the PGC data are based on clinical samples; as such these are known to have certain biases, such as inflated comorbidity, and decreased global functioning, compared to community derived samples. These different sample biases may act to decrease the ability to identify genetic associations between them. Third, PRS used in the current study were calculated based on results from case-control genome-wide association studies (GWAS) and not dimension-specific schizophrenia or bipolar disorder symptoms. It is possible that there would be greater predictive power on specific psychotic experiences from a PRS that was specific to each symptom grouping within schizophrenia or bipolar disorder. This is further supported by the evidence from a number of genetic association studies and a twin study, which show that symptom variation within clinical psychosis is partly influenced by ‘modifier genes’(genes that influence clinical features of a disease but not its liability) [38],[39], which might also influence adolescent psychotic experiences. However, because schizophrenia can include each of the psychotic experiences used in this study, it was still hypothesised that each specific psychotic experience would be predicted by the schizophrenia PRS. Fourth, evidence from one twin study suggests that while psychotic experiences are moderately stable across adolescence, new genetic influences become involved across age [13]. It is therefore plausible that the SNPs associated with schizophrenia and bipolar disorder that were tested in the current study are not yet involved at age 16. The higher prevalence of ‘confirmed’ psychotic experiences in adolescence than of psychotic disorders in adults suggests some psychotic experiences dissipate in individuals over time [27]. However, our hypothesis for an association between loci associated with schizophrenia and adolescent psychotic experiences is supported by phenotypic associations reported between earlier psychotic experiences and adult psychosis [4],[5]. Fifth, the genetic correlation between adolescent psychotic experiences and adult schizophrenia is not known because no twin data or genome-wide complex trait analysis (GCTA) [40],[41] findings on this bivariate relationship have been reported. It is not known the degree to which the relationship between adolescent psychotic experiences and psychotic disorders is influenced by genetic or environmental influences. Finally, as further PRS become available that have greater accuracy of prediction of their own phenotype (e.g. of schizophrenia or bipolar disorder), they will offer greater power for testing predictions about genetic relationships with related phenotypes such as psychotic experiences in younger individuals [42].

### Approaches 2 and 3: single SNP analyses and schizophrenia composite score

The single SNP analyses performed in Approach 2 of our study yielded two significant associations between previously identified loci for schizophrenia liability and Paranoia, one of the dimension-specific psychotic experiences. These signals were not replicated in an independent sample measured on a cumulative scale of positive psychotic experiences (not on Paranoia specifically). It is noteworthy that although not replicated, both SNPs (rs17512836 and rs9960767; *r* ^2^ = 0.44, D' = 0.95) identified in the validation sample were located in an intron of TFC4. It is possible that replication of Approach 2 findings in an independent sample failed because of the difference in measures used. We note that the two measures show a good agreement [14] but unlike SPEQ, PLIKS-Q (the measure of psychotic experiences used in the replication sample) is not a dimension-specific measure. Instead it includes a variety of positive psychotic experiences within a single scale.

In our data we observed a trend towards different SNPs showing stronger associations with some of the dimensions of psychotic experiences; although not replicated, these findings are consistent with genetic studies of schizophrenia symptoms. These suggest that some schizophrenia liability genes are more strongly associated with some of the schizophrenia symptoms than with others [43]–[46]. For example, SNPs in COMT have been shown to associate more strongly with manic symptoms [43] whilst SNPs in DTNBP1 with negative symptoms [45].

In the current study, when the 28 selected SNPs were aggregated into a composite schizophrenia SNP score in Approach 3, they yielded no significant results for any of the dimensions.

### Limitations

The results of our study should be interpreted in light of some limitations. First, it would have been ideal to have a mania subscale included in the measurement. Second, the measure used in the replication sample did not allow for specific psychotic experiences to be assessed individually. Third, self and parent reports of psychotic experiences are likely to include specific forms of measurement error due to circumstances of individuals misunderstanding the nature of the phenomena being asked about, and a lack of adjustment for cultural norms. Fourth, our sample size may have been a limiting factor in detection of the potential positive associations. It is noted that the one other study similar to this one in its aims had a validation sample that was double the size of this one and they were only just able to detect ‘very weak effects’ [27].

### Conclusions

The current study provides the first empirical test of whether aggregated common variants and single SNPs associated with schizophrenia and bipolar disorder are also predictive of dimension-specific psychotic experiences in adolescence. In order to improve on this existing work, future studies should aim to increase the sample sizes assessed on psychotic experiences and employ future polygenic risk scores that explain larger proportions of the liability to schizophrenia and bipolar disorder. Taking into account the transient nature of some psychotic experiences in adolescence is a challenge. Uncovering the genetic aetiology of psychotic experiences in adolescence could bring us a step closer to understanding the common pathway that takes people from experiencing psychotic experiences in adolescence to the point of developing clinically-recognised psychotic disorders. In future work, polygenic risk scores will hopefully offer the opportunity to predict, albeit in a limited and probabilistic way, groups of people at risk of complex heritable psychiatric illness [47].

## Supporting Information

File S1 **This file contains Tables S1-S20 and Figure S1.** Table S1, Number of SNPs per schizophrenia *p*-value threshold (P_T_). Table S2, Number of SNPs per bipolar disorder *p*-value threshold (P_T_). Table S3, Summary of selected SNPs. Table S4, TEDS SNP QC Results. Table S5, ALSPAC SNP QC Results. Table S6, Descriptive statistics for the schizophrenia polygenic risk scores across the eight *p*-value thresholds. Table S7, Descriptive statistics for the bipolar disorder polygenic risk scores across the eight *p*-value thresholds. Table S8, Summary of results for schizophrenia PRS as a predictor of SPEQ Paranoia subscale. Table S9, Summary of results for schizophrenia PRS as a predictor of SPEQ Hallucinations subscale. Table S10, Summary of results for schizophrenia PRS as a predictor of SPEQ Cognitive Disorganisation subscale. Table S11, Summary of results for schizophrenia PRS as a predictor of SPEQ Grandiosity subscale. Table S12, Summary of results for schizophrenia PRS as a predictor of SPEQ Anhedonia subscale. Table S13, Summary of results for schizophrenia PRS as a predictor of SPEQ Parent-rated Negative Symptoms subscale. Table S14, Summary of results for bipolar disorder PRS as a predictor of SPEQ Paranoia subscale. Table S15, Summary of results for bipolar disorder PRS as a predictor of SPEQ Hallucinations subscale. Table S16, Summary of results for bipolar disorder PRS as a predictor of SPEQ Cognitive Disorganisation subscale. Table S17, Summary of results for bipolar disorder PRS as a predictor of SPEQ Grandiosity subscale. Table S18, Summary of results for bipolar disorder PRS as a predictor of SPEQ Anhedonia subscale.Table S19, Summary of results for bipolar disorder PRS as a predictor of SPEQ Parent-rated Negative Symptoms subscale. Table S20, Full results of Allelic & Genotypic Association Analyses for Transformed Data (adjusted for sex and age). Figure S1, rs17512836 regional association plot for the Paranoia subscale scores. The figure was created with LocusZoom (http://csg.sph.umich.edu/locuszoom/). Mb, megabases.
(ZIP)
