# Supplementary material for: Are Genetic Risk Factors for Psychosis Also Associated with Dimension-Specific Psychotic Experiences in Adolescence?
Source: PLoS One. 2014 Apr 9;9(4):e94398. doi: 10.1371/journal.pone.0094398 (PMC3981778; doi:10.1371/journal.pone.0094398)
Supplement: File S1 — This file contains Tables S1-S20 and Figure S1. Table S1, Number of SNPs per schizophrenia p-value threshold (PT). Table S2, Number of SNPs per bipolar disorder p-value threshold (PT). Table S3, Summary of selected SNPs. Table S4, TEDS SNP QC Results. Table S5, ALSPAC SNP QC Results. Table S6, Descriptive statistics for the schizophrenia polygenic risk scores across the eight p-value thresholds. Table S7, Descriptive statistics for the bipolar disorder polygenic risk scores across the eight p-value thresholds. Table S8, Summary of results for schizophrenia PRS as a predictor of SPEQ Paranoia subscale. Table S9, Summary of results for schizophrenia PRS as a predictor of SPEQ Hallucinations subscale. Table S10, Summary of results for schizophrenia PRS as a predictor of SPEQ Cognitive Disorganisation subscale. Table S11, Summary of results for schizophrenia PRS as a predictor of SPEQ Grandiosity subscale. Table S12, Summary of results for schizophrenia PRS as a predictor of SPEQ Anhedonia subscale. Table S13, Summary of results for schizophrenia PRS as a predictor of SPEQ Parent-rated Negative Symptoms subscale. Table S14, Summary of results for bipolar disorder PRS as a predictor of SPEQ Paranoia subscale. Table S15, Summary of results for bipolar disorder PRS as a predictor of SPEQ Hallucinations subscale. Table S16, Summary of results for bipolar disorder PRS as a predictor of SPEQ Cognitive Disorganisation subscale. Table S17, Summary of results for bipolar disorder PRS as a predictor of SPEQ Grandiosity subscale. Table S18, Summary of results for bipolar disorder PRS as a predictor of SPEQ Anhedonia subscale.Table S19, Summary of results for bipolar disorder PRS as a predictor of SPEQ Parent-rated Negative Symptoms subscale. Table S20, Full results of Allelic & Genotypic Association Analyses for Transformed Data (adjusted for sex and age). Figure S1, rs17512836 regional association plot for the Paranoia subscale scores. The figure was created with LocusZoom (http: [file pone.0094398.s001.zip › Supplementary Information/Table S17.doc]

Table S17. Summary of results for bipolar disorder PRS as a predictor of SPEQ Grandiosity subscale

| p_T_ | β | 95% C.I. | t | *p*-value | R^2^ | N | df |
| --- | --- | --- | --- | --- | --- | --- | --- |
| .01 | -27.63 | -96.42-41.16 | -0.79 | 0.216 | .000 | 2160 | 9, 2150 |
| .05 | 12.91 | -129.97-155.77 | .018 | 0.430 | .000 |  |  |
| .10 | -97.23 | -293.26-98.81 | -0.97 | 0.166 | .000 |  |  |
| .20 | -91.80 | -370.82-187.22 | -0.65 | 0.260 | .000 |  |  |
| .30 | -146.12 | -469.47-204.23 | -0.82 | 0.207 | .000 |  |  |
| .40 | -198.88 | -611.25-213.50 | -0.95 | 0.172 | .000 |  |  |
| .50 | -243.03 | -712.87-226.82 | -1.01 | 0.156 | .000 |  |  |
| All | -323.68 | -982.98-335.63 | -0.96 | 0.168 | .000 |  |  |

Note. p_T_, p-value threshold; β, unstandardized beta value; C.I., confidence intervals; t, t-statistic; N, sample size; df, degrees of freedom; SPEQ Grandiosity subscale was log transformed; 8 PCs were included as covariates.
