# Supplementary material for: Are Genetic Risk Factors for Psychosis Also Associated with Dimension-Specific Psychotic Experiences in Adolescence?
Source: PLoS One. 2014 Apr 9;9(4):e94398. doi: 10.1371/journal.pone.0094398 (PMC3981778; doi:10.1371/journal.pone.0094398)
Supplement: File S1 — This file contains Tables S1-S20 and Figure S1. Table S1, Number of SNPs per schizophrenia p-value threshold (PT). Table S2, Number of SNPs per bipolar disorder p-value threshold (PT). Table S3, Summary of selected SNPs. Table S4, TEDS SNP QC Results. Table S5, ALSPAC SNP QC Results. Table S6, Descriptive statistics for the schizophrenia polygenic risk scores across the eight p-value thresholds. Table S7, Descriptive statistics for the bipolar disorder polygenic risk scores across the eight p-value thresholds. Table S8, Summary of results for schizophrenia PRS as a predictor of SPEQ Paranoia subscale. Table S9, Summary of results for schizophrenia PRS as a predictor of SPEQ Hallucinations subscale. Table S10, Summary of results for schizophrenia PRS as a predictor of SPEQ Cognitive Disorganisation subscale. Table S11, Summary of results for schizophrenia PRS as a predictor of SPEQ Grandiosity subscale. Table S12, Summary of results for schizophrenia PRS as a predictor of SPEQ Anhedonia subscale. Table S13, Summary of results for schizophrenia PRS as a predictor of SPEQ Parent-rated Negative Symptoms subscale. Table S14, Summary of results for bipolar disorder PRS as a predictor of SPEQ Paranoia subscale. Table S15, Summary of results for bipolar disorder PRS as a predictor of SPEQ Hallucinations subscale. Table S16, Summary of results for bipolar disorder PRS as a predictor of SPEQ Cognitive Disorganisation subscale. Table S17, Summary of results for bipolar disorder PRS as a predictor of SPEQ Grandiosity subscale. Table S18, Summary of results for bipolar disorder PRS as a predictor of SPEQ Anhedonia subscale.Table S19, Summary of results for bipolar disorder PRS as a predictor of SPEQ Parent-rated Negative Symptoms subscale. Table S20, Full results of Allelic & Genotypic Association Analyses for Transformed Data (adjusted for sex and age). Figure S1, rs17512836 regional association plot for the Paranoia subscale scores. The figure was created with LocusZoom (http: [file pone.0094398.s001.zip › Supplementary Information/Table S3.doc]

Table S3. Summary of selected SNPs

| SNP | Gene/Region | Chr | Published *p*-value | Reference | Other reference |
| --- | --- | --- | --- | --- | --- |
| rs1625579 | MIR137 | 1 | 1.59x10^-11^ | [1] |  |
| rs10489202 | BRP44 | 1 | 9.50x10^-9^ | [2] |  |
| rs2312147 | VRK2 | 2 | 1.90x10^-9^ | [3] |  |
| rs1344706 | ZNF804A | 2 | 2.50x10^-11^ | [4] |  |
| rs2239547^1^ | ITIH3-ITIH4 region | 3 | 7.83x10^-9^ | [1] |  |
| rs6904071 | MHC region | 6 | 1.78x10^-8^ | [5] |  |
| rs6913660 | MHC region | 6 | 1.10x10^-9^ | [6] | *p*=2.36x10^-8^ [5] & *p*=1.7x10^-11^ [3] |
| rs13194053 | MHC region | 6 | 9.50x10^-9^ | [7] | *p*=9.54x10^-9^ [5] |
| rs926300 | MHC region | 6 | 1.06x10^-8^ | [5] |  |
| rs13219354 | MHC region | 6 | 1.30x10^-10^ | [6] |  |
| rs13219181 | MHC region | 6 | 1.29x10^-8^ | [5] |  |
| rs6932590 | MHC region | 6 | 1.40x10^-12^ | [6] | *p*=1.60x10^-10^ [3] |
| rs3800316^2^ | MHC region | 6 | 3.81x10^-8^ | [5] |  |
| rs13211507 | MHC region | 6 | 8.30x10^-11^ | [6] | *p*=1.4x10^-13^ [3] |
| rs1233710 | ZKSCAN4/MHC region | 6 | 4.76x10^-11^ | [8] |  |
| rs1635 | NKAPL/MHC region | 6 | 6.91x10^-12^ | [8] |  |
| rs2021722 | TRIM26/MHC region | 6 | 2.18x10^-8^ | [1] |  |
| rs3131296 | MHC region | 6 | 2.30x10^-9^ | [6] | *p*=5.1x10^-11^ [3] |
| rs10503253 | CSMD1 | 8 | 4.14x10^-8^ | [1] |  |
| rs16887244 | LSM1 | 8 | 1.27x10^-9^ | [2] |  |
| rs1488935 | WHSC1L1 | 8 | 5.06x10^-9^ | [2] |  |
| rs7004633 | MMP16 | 8 | 2.75x10^-8^ | [1] |  |
| rs10994359^1^ | ANK3 | 10 | 2.45x10^-8^ | [1] |  |
| rs7914558 | CNNM2 | 10 | 1.82x10^-9^ | [1] |  |

Table S3. Cont.

| SNP | Gene | Chr | Published *p*-value | Reference | Other reference |
| --- | --- | --- | --- | --- | --- |
| rs11191580 | NT5C2 | 10 | 1.11x10^-8^ | [1] |  |
| rs835784 | TSPAN18 | 11 | 2.73x10^-11^ | [8] |  |
| rs12807809 | NRGN | 11 | 2.40x10^-9^ | [6] | *p*=2.80x10^-9^ [3] |
| rs548181 | STT3A | 11 | 2.91x10^-8^ | [1] |  |
| rs4765905^1^ | CACNA1C | 12 | 7.01x10^-9^ | [1] |  |
| rs4309482 | TCF4/CCDC68 | 18 | 7.80x10^-9^ | [3] |  |
| rs12966547 | CCDC68 | 18 | 2.60x10^-10^ | [1] |  |
| rs17512836 | TCF4 | 18 | 2.35x10^-8^ | [1] |  |
| rs9960767 | TCF4 | 18 | 4.10x10^-9^ | [6] | *p*=4.20x10^-9^ [3] |

Note. *SNP*, single nucleotide polymorphism; *Chr*, chromosome; *Reference*, reference for the *p*-valued and risk allele used in the current study; *Other reference*, reference and the *p*-value for the same SNP reported elsewhere; ^1^ SNPs significant based on joint analysis of schizophrenia and bipolar disorder; ^2^ in the analysis rs380016 was used as a proxy for rs926300.

**References**

1.	Ripke S, Sanders AR, Kendler KS, Levinson DF, Sklar P, et al. (2011) Genome-wide association study identifies five new schizophrenia loci. Nat Genet 43: 969–976.

2.	Shi Y, Li Z, Xu Q, Wang T, Li T, et al. (2011) Common variants on 8p12 and 1q24.2 confer risk of schizophrenia. Nat Genet 43: 1224–1227.

3.	Steinberg S, de Jong S, Andreassen OA, Werge T, Børglum AD, et al. (2011) Common variants at VRK2 and TCF4 conferring risk of schizophrenia. Hum Mol Genet 20: 4076–4081.

4.	Williams HJ, Norton N, Dwyer S, Moskvina V, Nikolov I, et al. (2011) Fine mapping of ZNF804A and genome-wide significant evidence for its involvement in schizophrenia and bipolar disorder. Mol Psychiatry 16: 429–441.

5.	Shi J, Levinson DF, Duan J, Sanders AR, Zheng Y, et al. (2009) Common variants on chromosome 6p22.1 are associated with schizophrenia. Nature 460: 753–757.

6.	Stefansson H, Ophoff RA, Steinberg S, Andreassen OA, Cichon S, et al. (2009) Common variants conferring risk of schizophrenia. Nature 460: 744–747.

7.	Purcell SM, Wray NR, Stone JL, Visscher PM, O’Donovan MC, et al. (2009) Common polygenic variation contributes to risk of schizophrenia and bipolar disorder. Nature 460: 748–752.

8.	Yue W-H, Wang H-F, Sun L-D, Tang F-L, Liu Z-H, et al. (2011) Genome-wide association study identifies a susceptibility locus for schizophrenia in Han Chinese at 11p11.2. Nat Genet 43: 1228–1231.
